# Supplementary material for: A Physiologically Based Pharmacokinetic Model to Predict Determinants of Variability in Epirubicin Exposure and Tissue Distribution
Source: Pharmaceutics. 2023 Apr 12;15(4):1222. doi: 10.3390/pharmaceutics15041222 (PMC10143085; doi:10.3390/pharmaceutics15041222)
Supplement: Supplementary file 1 [file pharmaceutics-15-01222-s001.zip › pharmaceutics-2147854-supplementary.pdf]

Supplemental Data

Supplemental Table S1: The mean values and range of all physiological characteristics used in the simple linear regression analysis model. 2000 Sim-Cancer patients were generated in this study.

**Physiological Parameter**	**Mean**	**Std Dev**	**5th centile**	**95th centile**	**P Value (do values differ from 0)**
Age (Years)	66.79	14.04	42.73	88.83	<0.0001
Weight (kg)	70.60	14.09	50.32	95.27	0.004
Height (cm)	165.62	9.73	149.97	181.88	0.0035
BSA (m^2)	1.78	0.20	1.47	2.11	0.0014
BMI (kg/m^2)	25.69	4.41	19.36	33.16	0.2211
Haematocrit (%)	37.64	4.63	30.28	45.70	<0.0001
Albumin (g/L)	38.18	6.90	27.76	49.99	<0.0001
GFR (mL/min/1.73 m^2)	79.80	23.62	47.61	123.08	<0.0001
Albumin (g/L)	38.18	6.905	27.72	50.01	0.0014
LiverUGT2B7 (pmol)	2965937	1512899	1143503	5901583	<0.0001
GutUGT2B7 (pmol)	13751	9171	3974	31676	0.7856
KidneyUGT2B7 (pmol)	177510	162817	41202	466093	<0.0001

Supplemental Table S2: Mean and range enzymatic expression of UGTs incorporated into the model.

Enzyme	Mean (pmol)	Std Dev	5th centile	95th centile
Liver				
UGT1A1	1685500.93	993902.51	500913.21	3612128.83
UGT1A3	990058.04	566915.04	371500.68	2091593.26
UGT1A4	2202537.56	1079931.96	967923.39	4148718.19
UGT1A5	0.00	0.00	0.00	0.00
UGT1A6	840286.24	422171.05	342314.82	1646158.96
UGT1A7	0.00	0.00	0.00	0.00
UGT1A8	0.00	0.00	0.00	0.00
UGT1A9	1260309.51	713121.23	351967.04	2557354.27
UGT1A10	848.42	424.01	334.97	1651.86
UGT2B4	2263846.38	1110017.02	959048.99	4423238.59
UGT2B7	2965937.20	1512898.54	1143637.73	5894268.63
UGT2B10	276816.45	141780.73	107524.68	536167.74
UGT2B11	0.00	0.00	0.00	0.00
UGT2B15	754277.95	750628.57	62731.22	2139367.98
UGT2B17	153075.36	141330.66	15303.50	417525.92
UGT2B28	0.00	0.00	0.00	0.00
Kidney				
UGT1A1	18873.94	18302.13	3524.75	54716.17
UGT1A3	0.00	0.00	0.00	0.00
UGT1A4	31972.12	27400.22	6617.03	83472.37
UGT1A5	0.00	0.00	0.00	0.00
UGT1A6	13254.10	11197.18	2818.93	34990.87
UGT1A7	48286.99	43588.42	10040.55	129462.17

UGT1A8	18093.15	15089.74	3964.87	46689.78
UGT1A9	273796.48	247215.96	41429.33	742289.63
UGT1A10	62582.66	58719.85	13084.64	166188.26
UGT2B4	1134.54	1038.68	244.67	3012.61
UGT2B7	177510.02	162816.84	41241.74	465485.96
UGT2B10	0.00	0.00	0.00	0.00
UGT2B11	0.00	0.00	0.00	0.00
UGT2B15	346.23	446.85	17.86	1154.06
UGT2B17	0.00	0.00	0.00	0.00
UGT2B28	0.00	0.00	0.00	0.00

Supplemental Table S3: Multivariate linear regression analysis of the model predicted variables affecting epirubicin LnAUC and their respective linearity regarding LnAUC with the exclusion of BSA. R^2 of the model is 0.8324.

**Variable**	**Estimated Ln AUC (ng/mL.h)**	**Standard error**	**Range (95% CI)**	**R^2 with other variables**	**P value**
Intercept	8.556	0.02954	8.498 to 8.614		<0.0001
Sex[0]	-0.07619	0.003993	-0.08402 to -0.06836	0.05004	<0.0001
Age	0.003195	0.0001937	0.002816 to 0.003575	0.4880	<0.0001
Haematocrit	-0.005730	0.0004210	-0.006556 to -0.004905	0.003825	<0.0001
Albumin	0.01125	0.0002840	0.01069 to 0.01180	0.01507	<0.0001
GFR	-0.0007297	0.0001087	-0.0009428 to -0.0005166	0.4251	<0.0001

Liver UGT2B7	-9.052e-008	1.476e-009	-9.342e-008 to -8.763e-008	0.2403	<0.0001
Kidney UGT2B7	-2.959e-007	1.206e-008	-3.196e-007 to -2.723e-007	0.01765	<0.0001

Model variables; sex 0=Female, 1=Male, age (years), haematocrit (%), albumin (g/L), glomerular filtration rate (GFR) (mL/min/1.73m^2), liver UGT2B7 (pmol), kidney UGT2B7 (pmol).
